# Cytomegalovirus Infections in Hematopoietic Stem Cell Transplant: Moving Beyond Molecular Diagnostics to Immunodiagnostics

**DOI:** 10.3390/diagnostics14222523

**Published:** 2024-11-12

**Authors:** Chhavi Gupta, Netto George Mundan, Shukla Das, Arshad Jawed, Sajad Ahmad Dar, Hamad Ghaleb Dailah

**Affiliations:** 1 Department of Infectious Diseases, Yashoda Super Speciality Hospital, Ghaziabad 201001, India; 2 Department of Infectious Diseases, Government Medical College, Kottayam 686008, India; 3 Department of Microbiology, University College of Medical Sciences and GTB Hospital (University of Delhi), Delhi 110095, India; 4 College of Nursing and Health Sciences, Jazan University, Jazan 45142, Saudi Arabiasdar@jazanu.edu.sa (S.A.D.)

**Keywords:** cytomegalovirus, immune reconstitution, immune monitoring, prophylaxis, pre-emptive

## Abstract

Human CMV, regularly reactivated by simple triggers, results in asymptomatic viral shedding, powerful cellular immune responses, and memory inflation. Immunocompetent individuals benefit from a robust immune response, which aids in viral management without causing clinically significant illness; however, immunodeficient individuals are always at a higher risk of CMV reactivation and disease. Hematopoietic stem cell transplant (HSCT) recipients are consistently at higher risk of CMV reactivation and clinically significant CMV illness due to primary disease, immunosuppression, and graft vs. host disease. Early recovery of CMV-CMI responses may mitigate effects of viral reactivation in HSCT recipients. Immune reconstitution following transplantation occurs spontaneously and is mediated initially by donor-derived T cells, followed by clonal growth of T cells produced from graft progenitors. CMV-specific immune reconstitution post-transplant is related to spontaneous clearance of CMV reactivation and may eliminate the need for prophylactic or pre-emptive medication, making it a potential predictive marker for monitoring CMV reactivation. This review highlights current thoughts and therapeutic options for CMV reactivation in HSCT, with focus on CMV immune reconstitution and post-HSCT monitoring. Immune monitoring aids in risk stratification of transplant recipients who may progress from CMV reactivation to clinically significant CMV infection. Implementing this approach in clinical practice reduces the need for periodic viral surveillance and antiviral therapy in recipients who have a high CMV-CMI and thus may experience self-limited reactivation. Therefore, in the age of precision medicine, it is critical to incorporate CMV-specific cellular immune surveillance into conventional procedures and algorithms for the management of transplant recipients.

## 1. Introduction

Human cytomegalovirus (CMV), belonging to the genus Cytomegalovirus and the family Herpesviridae, is species-specific and exhibits broad cellular tropism, with latency being a characteristic feature. HCMV infects many cell types (particularly in lytic infection) but latent infection appears to be maintained in CD34 cells and CD33 early myeloid cell progenitors predominantly and also in endothelial cells, neuronal progenitor cells, and fibroblasts [1,2]. Even with trivial triggers (like common cold or flu, allergies, insufficient sleep patterns, minor injuries or wounds, dehydration, unsanitary practices, environmental impacts, dietary inadequacies, emotional stress, etc.) which might not directly cause CMV reactivation but can contribute to a weakened immune system or create an environment conducive to reactivation when combined with other factors, the periodic burst of CMV reactivation and asymptomatic shedding of the virus is observed in blood and body fluids. Robust immune responses to CMV, particularly memory T cells, help in restricting CMV replication in immunocompetent persons, however, in conditions with attenuated T cell responses like prolonged corticosteroid therapy, human immunodeficiency virus infection, transplant settings, and T-cell-depleting therapies, the immune system might not be able to control CMV replication, manifesting as clinically significant CMV infections (CS-CMVi) [3]. CMV-specific T cell responses are involved in a dynamic, life-long interaction between the host and virus; T cells, though essential for restricting viral replication, do not completely eradicate the virus from the human body and continued but suppressed viral replication helps generate a pool of memory cells. Interestingly, accumulation of CMV-specific T cells occurs with increasing age due to periodic activation of latent virus inducing stronger secondary immune responses, generating memory T cells, a phenomenon termed as memory inflation [4,5]. This review summarizes the current concepts of CMV reactivation in HSCT and management strategies with special attention to CMV immune reconstitution and immune monitoring post-HSCT.

### 1.1. CMV in HSCT

In HSCT recipients, primary disease, conditioning regimens, immunosuppressive agents, and graft versus host disease (GVHD) result in cellular and humoral immune system deficiencies [6]. The recovery of the immune system starts with engraftment 2–4 weeks after stem cell infusion and full immune recovery takes approximately two years [6]. The immunosuppressed HSCT recipients are at increased risk of infections, either donor-derived, or due to reactivation of latent infections, or acquired exogenously. CMV infection and disease in transplant settings are primarily due to CMV reactivation and rarely due to de novo infection. Following HSCT, the incidence of CMV reactivation has been documented to range from 30% to 70%. This could be associated with an elevated non-relapse mortality rate, estimated to range from 45% to 60% [7]. In HSCT, the risk of CMV reactivation and disease is higher when recipients are seropositive (CMV immunoglobulin G (IgG) positive; for R^+^ status). The risk of reactivation increases to 60% in R^+^ in the presence of GVHD, and to approximately 10% in seronegative R recipients (R^−^) transplanted from seropositive donors (D^+^) [8]. Other risk factors include human leucocyte antigen (HLA) mismatch, advanced age, other concurrent infections (such as with herpes virus 6 or 7), genetic polymorphisms, and conditioning regimen used. The use of lymphocyte-depleting antibodies such as antithymocyte globulin or T-cell-depleting therapies like alemtuzumab is associated with greater risk while mTOR inhibitors have a low risk of reactivation [9,10].

The onset of CMV reactivation can occur at any time after engraftment up to 100 days post-transplant, which is the high-risk period; patients may present CMV syndrome characterized by febrile illness or CMV-CSi or tissue-invasive disease presenting as colitis or pneumonitis, hepatitis, encephalitis, retinitis, nephritis, pancreatitis, or myocarditis [11]. In addition, CMV due to low-level persistence and immunomodulating properties can also result in indirect effects such as transplant vasculopathy, tubulointerstitial fibrosis, and bronchiolitis obliterans leading to grave consequences of acute or chronic graft rejection [12,13]. Further, it may predispose the recipient to develop other opportunistic infections, particularly invasive bacterial and fungal diseases like *Nocardia* spp., *Aspergillus* spp., and herpes viruses [9].

### 1.2. CMV Diagnosis

Conventional diagnostic modalities for CMV detection are phosphoprotein (pp65) antigen detection or viral culture. Current diagnostic modalities include serology (antibody detection) and viral detection by polymerase chain reaction (PCR). Each diagnostic modality has its advantages and disadvantages [14,15] (Table 1).

### 1.3. CMV Management Strategies in HSCT

**Figure 1:**
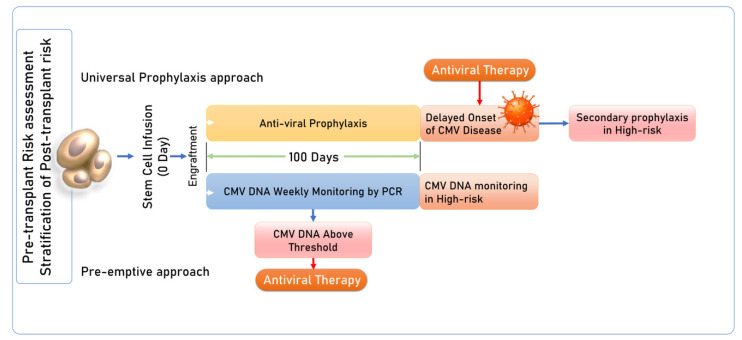
Conventional prophylaxis and pre-emptive strategies for CMV reactivation in HSCT recipients. (1) Universal prophylaxis approach: Primary prophylaxis with antivirals initiated in HSCT recipients, post-cessation of primary prophylaxis there may be late-onset CMV reactivation or disease; after treatment, secondary prophylaxis with antivirals is started again. (2) Pre-emptive approach: Periodic monitoring of viral DNA by PCR, followed by antiviral therapy for DNAemia [16,17,18,19,20].

The current strategy for CMV management involves antiviral prophylaxis or pre-emptive therapy (Figure 1). The goal of prophylactic therapy involves the use of drugs before the onset of viral replication while pre-emptive therapy utilizes the strategy of routine viral surveillance by CMV DNA monitoring and pre-emptive treatment based upon viremia to prevent the progression of CMV DNAemia to tissue-invasive disease [16] (Figure 1). Earlier antiviral prophylaxis involved the use of the nucleoside analogues ganciclovir, valganciclovir, and cidofovir. However, due to drug interactions and associated side effects, the focus for management has been on standard pre-emptive therapy. This includes CMV DNA monitoring, which is conducted at least once a week in the first 100 days post-transplant, once every other week in the following 3 months, and once every month until GVHD prophylaxis withdrawal. Frequent monitoring may be necessary for high-risk recipients, such as those undergoing cord blood transplant, receiving transplants from haploidentical or matched unrelated donors, or those experiencing active GVHD, especially in D^−^/R^+^ seropositive recipients [16,17,18]. The viral load cut-off for pre-emptive therapy is still a matter of debate as no consensus has been established, but most of the guidelines recommend pre-emptive therapy when the level of CMV DNA is more than 1000 copies per millimeter (/mL) in plasma or 10,000 copies/mL in whole blood in two consecutive assessments [18]. CMV viral load significantly differs in plasma and whole blood and there may be intra-assay variability in the same sample (either plasma or whole blood) and the same commercial system should be used for sequential reliable monitoring. The guidelines also recommend that every transplant center establish its own cut-off. However, this approach suffers from certain limitations like viral blips and low or undetectable CMV viral load in blood samples in the event of compartmentalized disease like CMV colitis. Also, not all episodes of CMV reactivation may progress to clinically significant disease and antivirals are associated with side effects of myelosuppression and renal toxicity, further complicating the course of transplant. With the recent introduction of letermovir and the literature proving its beneficial role with lesser toxicity and fewer drug interactions, many centers have opted for a prophylactic strategy using letemorvir in high-risk patients as both primary and secondary prophylaxis [19,20].

### 1.4. CMV Immune Reconstitution and Immune Monitoring in HSCT

Immune reconstitution (IR) post-transplantation is a unique multidimensional process depending on the source of stem cells, human leukocyte antigen (HLA) barriers, conditioning regimen used, and post-transplantation interventions. The transfused stem cells act as reservoirs of immune cells and initiate the complex process of IR post-transplant [21]. In the first phase, clonal expansion of naïve and antigen-experienced T cells presents in the graft, termed as homeostatic peripheral expansion (HPE), which is cytokine-dependent and occurs particularly in CD8^+^ T cells with a limited repertoire resulting in early but incomplete immune responses. The TCR repertoire during this phase is usually dominated by expansion of singular clonotypes. This step depends upon donor age (the ratio of naïve T cells to memory T cells) and the type of graft (bone marrow vs. PBSCs), which determines the quantity of regenerating T cell numbers, while recipient age influences the duration of this period. The second phase involves the differentiation of donor-derived hematopoietic precursors schooled in the recipient’s bone marrow or thymus which is the mechanism for immunological reconstitution following HSCT and results in long-lasting immune responses with a diverse T cell repertoire [5,21,22]. In the final phase, components of innate as well as adaptive immunity achieve a balanced and stable immune system. In the final phase, components of both innate and adaptive immunity reach a balanced and stable state, relative to the patient’s age, and the diversity of the TCR repertoire is polyclonal at this stage [21].

Early recovery of CMV-CMI responses can offset the consequences of viral reactivation in HSCT recipients [20,21,22,23]. Post-HSCT, cellular immune responses spontaneously recover, and the numbers of CD4^+^ and CD8^+^ cells are associated with the resolution of CMV reactivation [23]. As a result, T cell response may be a key predictor of CMV infection after transplantation. Absolute numerical recovery of CMI is insufficient; concurrent functional recovery in terms of cytokine production and establishment of long-term memory cells is also necessary [24,25]. The immune recovery process after transplantation depends on continuous antigenic stimulation via viral reactivation for the clonal proliferation of CMV-specific T cells, which is mediated in the early transplant period by donor-derived T cells and later by clonal expansion of T cells derived from graft progenitors [26,27]. Recent research suggests that in recipients with reduced intensity conditioning, recipient immune cells may contribute to immunity before chimerism with donor cells [27,28,29].

## 2. Role of Genetic Polymorphism

There is emerging evidence of genetic polymorphisms in the immune molecules involved in the CMV–host interaction, influencing the reactivation and natural history of CMV infection post-transplant [30,31,32]. Studies have identified that single-nucleotide polymorphisms (SNPs) of genes coding for cytokines or chemokines and their receptors or involved in innate immunity, such as Toll-like receptors 2, 4, mannose-binding lectin, interferon lambda, etc., may influence the dynamics of gene transcription, the stability of mRNA, and production and biological activity of the resulting cytokine or chemokine, thus influencing the susceptibility to CMV reactivation or tissue-invasive disease [30,31,32]. It has also been observed that such SNPs in both the donor and recipient may affect susceptibility to GVHD indirectly, also affecting CMV dynamics [32]. For instance, Vallejo et al. studied genetic polymorphism in both donors and recipients in allogenic HSCT and also made a risk prediction model for CMV reactivation and risk stratification [30]. They identified 11 polymorphisms in seven different genes (CXCL12, IL12A, KIR3DL1, TGFB2, TNF, IL1RN, and CD48) that were associated with either development or protection from CMV reactivation irrespective of CMV serostatus. The TNF-α SNP rs3093662 (GA/GG) genotype in donors and recipients was associated with protection from CMV infection after allo-HSCT. In addition, genetic variations of CD48 rs2295615 (GC/GG) and IL1RN rs439154 (AG/AA) in the donor were associated with protection from CMV reactivation while the CXCL12 rs2839695 (GG/AG) SNP and *KIR3DL1* rs45542639 (AG/AA), rs149123986 (GA/GG), rs144994606 (AG/AA), and rs143159382 (TC/TT) in the recipient were associated with an increased risk of CMV infection. The authors made a genetic risk predictive score model with five genetic polymorphisms (*CXCL12* rs2839695, *IL12A* rs7615589, *KIR3DL1* rs4554639, *TGFB2* rs5781034 for the recipient and *CD48* rs2295615 for the donor) and defined a cut-off of 0.49 to stratify high-risk and low-risk patients for CMV reactivation. In another study, Campos et al. demonstrated a significant association between genetic variation in pentraxin 3 (PTX3) and CMV reactivation [32]. For the donor SNP rs2305619, risk of CMV reactivation was higher for the GG genotype than AG and AA (56%, 38%, and 36%, respectively, *p* value 0.01); while for rs3816527, the cumulative incidence of CMV infection was 55% for AA, 32% for AC, and 37% for CC genotypes (*p* = 0.03). Further, h2/h2 (G-A, G-A) haplotype donors were significantly associated with CMV reactivation while the recipient’s haplotype did not influence CMV reactivation. In this study, this genetic polymorphism effect was also independent of the CMV serostatus of recipients. However, genetic polymorphism studies are in a very nascent stage and more literature is required to derive any conclusion. Results about genetic polymorphisms cannot be extrapolated to all patients considering variations in the genetic make-up of different ethnic populations.

## 3. Method for Immune Monitoring

**Figure 2:**
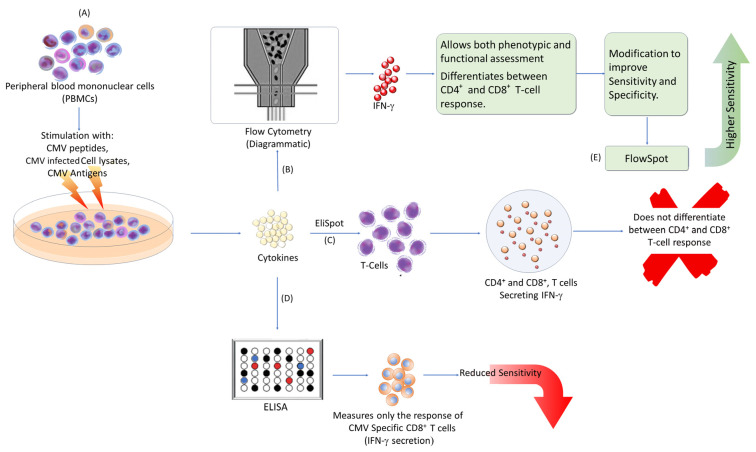
Various methods for immune monitoring. (**A**) Peripheral blood mononuclear cells (PBMCs) from recipients are separated, and naïve T cells are then stimulated with CMV peptides, infected cell lysates, or CMV antigens. Stimulated T cells produce IFN-γ. (**B**) CMV-specific CD4^+^ and CD8^+^ T cells quantified and secreted IFN-γ measured by flow-cytometry-based assays. (**C**) CMV-specific CD4^+^ and CD8^+^ T cells secreting IFN-γ are captured and measured at single cell level by spot-forming cells. (**D**) CMV-specific CD8^+^ T cells release IFN-γ measured by ELISA-based assay. (**E**) The sensitivity of flow-cytometry-based assays improved by using capturing beads in the FlowSpot test [6,33,34,35,36,37,38,39,40].

Current diagnostic methods for assessing CMV-specific immune responses typically involve ex vivo stimulation of naive T cells with CMV peptides or lysates from infected cells. The immune responses are then measured using enzyme-linked immunosorbent assay (ELISA), enzyme-linked immunosorbent spot (ELISpot), or flow cytometry-based assay (FACS) [6,33,34,35,36,37]. These methods are summarized in Figure 2.

The ELISA-based assay, often referred to as interferon-γ (IFN-γ)-releasing assay (IGRA) or QuantiFERON assay, measures the release of IFN-γ by stimulated CD8^+^ T cells. Blood is incubated with CMV peptides, and the released cytokines are measured by ELISA. While these tests are easy to perform, they have reduced sensitivity and only measure CMV-specific CD8^+^ T cell responses. Additionally, intermediate results can be difficult to interpret [33,34,35].

ELISpot quantifies IFN-γ released by both CD4^+^ and CD8^+^ T cells stimulated with CMV-specific peptides. It counts the number of spots formed by cytokine-releasing cells (SFCs) per a given number of target cells. This method is highly reproducible and provides precise results but does not differentiate between CD4^+^ and CD8^+^ T cell immune responses [36,37,38,39].

Flow cytometry involves stimulating cells with CMV antigens, followed by detecting intracellular cytokines and cell surface markers in real time using fluorescein antibodies. This method can differentiate between CD4^+^ and CD8^+^ T cells and allows for both phenotypic and functional assessments of CMV-reactivated cells [6,23,33,39]. Although more sensitive than ELISA-based assays, flow cytometry tests are challenging to standardize.

Several modifications have been made to enhance the sensitivity and specificity of these methods. For example, FlowSpot combines peripheral blood mononuclear cells with IFN-γ capture beads. When T cells are stimulated with CMV peptides, they release cytokines that are captured by nearby beads. The relative number of CMV-specific T cells and their cytokine release activity are measured using flow cytometry analysis [40]. FlowSpot has been shown to be more sensitive than ELISpot and requires less execution time [40].

Further improvements in assay performance can be achieved by carefully selecting CMV antigenic peptides for stimulation. Options include urea-formulated or T-activated proteins or using fluorescent-labeled multimers of MHC–peptide complexes in Dextramer assays [41,42].

## 4. Utility of Immune Monitoring in HSCT

Numerous studies have assessed CMV-CMI responses measured serially at regular intervals to determine the risk of CMV infection and disease after HSCT. These studies have concluded that the reconstitution of CMV-specific CMI is associated with the spontaneous clearance of CMV reactivation. Thus, it may serve as a predictive marker for monitoring CMV reactivation and could potentially obviate the need for prophylaxis or pre-emptive therapy [24,34,35,36,37,38,39] (Table 2).

The presence of CMV-specific CMI after the first episode of reactivation may predict future reactivation episodes. A study evaluated CMV-specific immunity after the end of treatment for the first episode of CMV reactivation and found that following the initial CMV reactivation, patients who exhibited positive QF-CMV results experienced no recurrent infections thereafter. In contrast, a significant proportion of patients with indeterminate or negative results had recurrent CMV infections, thus establishing the role of CMV-specific T cell immunity in predicting recurrent reactivations [35].

The role of CMV-specific immune reconstitution for spontaneous clearance of viral reactivation has been well proven but there is no protocol regarding the endpoints for measurement of CMI. In most of these studies, assays for CMI (like CMV IGRA, CMV ELISpot, or intracellular cytokine staining) were performed weekly or monthly after engraftment until 100 days or 12 months from transplant. The minimal number of CD3^+^ T cells required for accurate results after engraftment has not been established yet, but some laboratories have established a minimum of 100 T lymphocytes per cubic millimeter of blood for specific immune responses [17]. In an observational multicentric, prospective study by Yong et al., CMV-specific T cell immunity was assessed at baseline and then quarterly up to 12 months after transplant using CMV IGRA, CMV ELISpot, and intracellular cytokine staining methods [43]. The sensitivity of the CMV IGRA assay at an IFN-γ cut-off of 0.2 IU/mL was 74%, which improved to 86% at a lower cut-off of 0.1 IU/mL, with specificity being 100% at both cut-offs. The sensitivity and specificity of the CMV ELISpot were 98% and 44%, respectively, relative to CMV serology prior to transplant [43]. In another study by Krawczyk et al., the threshold of 8.9 IU/mL (specificity—100%; sensitivity—42%) correlates with protection from high-level viremia (defined as 5000 copies/mL in this study) [44].

## 5. Functional Immune Monitoring

It has been seen that cellular immune recovery has an inverse relationship with the risk of viral reactivation. Currently, many transplant centers are focused only on phenotypic assessment of T cell subsets post-transplant while some transplant groups have sought to incorporate functional immune monitoring to assess the functional capacity of reconstituting cells concerning CMV [39,43,45]. Yong et al. showed that the presence of dual functional T cells (IFN-γ^+^/TNF-α^+^) in recipients was not associated with CMV reactivation [43], while Krol et al. showed that IFN-γ^+^/IL2^+^ CD8^+^ T cells were associated with better CMV viral control after HSCT, thus concluding that polyfunctional antigen-specific T cells are better able to control viral replication [45].

In a recent landmark study, Naik et al. demonstrated an absolute threshold of 300 CD3^+^ T cells, above which functional virus-specific immunity exists, resulting in spontaneous viral clearance [39]. The authors tracked T cell recovery quantitatively using flow cytometry and qualitatively by IFN-γ ELISpot using viral antigenic peptides as a stimulus along with viral surveillance for up to 1-year post-transplant in a cohort of 23 pediatric allogeneic HSCT recipients. The study highlighted that viral reactivation was the main driver of functional virus-specific T cell reconstitution, and those with reactivations exhibited robust CMV-directed responses while those without CMV reactivations demonstrated only baseline antigen-specific activity. On further comparison with absolute CD3^+^ T cells with CMV-specific immunity, recipients with CD3^+^ T cell counts >300 cells per cubic millimeter exhibited potent and functionally protective levels of CMV-directed T cell activity while in recipients with a CD3^+^ T cell count less than 300 cells, CMV-specific immunity varied from non-functional activity to strong activity. Further, it was also observed that T-cell-depleted transplants exhibited a delay in quantitative immune reconstitution while qualitative immune reconstitution was comparable with non-T-cell-depleted counterparts. However, due to the small cohort of pediatric transplants, extrapolation of results to clinical practice for the adult population is not possible due to varying immune competences, age-related thymic changes, and the difference in conditioning regimens or GVHD prophylaxis. The study by Naik et al. [39] provides a novel concept of phenotypic and functional immune monitoring for personalized management of post-transplant infections.

The role of adoptive transfer of CMV-specific T cells in treating CMV infections that do not respond to antiviral treatment has been investigated [46]. Although the study is focused on treatment, its findings have major significance for immunological surveillance of CMV in HSCT recipients.

Extensive CMV management guidance for HSCT recipients is provided in the study of Boeckh and Ljungman [47]. The study does not directly address immunological surveillance, but it emphasizes its importance in CMV control in HSCT recipients. They evaluated clinical immune-monitoring methods like CMV-specific T cell assays (e.g., ELISpot, intracellular cytokine staining) to predict CMV reactivation, guide pre-emptive therapy, and assess antiviral treatment response. They also addressed immune-monitoring issues in HSCT recipients, such as assay sensitivity and specificity variability, interpretation of results in the context of immunosuppressive therapy, and the need for standardized immune monitoring in clinical practice.

Assessing CMV-specific T cell responses helps detect CMV reactivation and initiate pre-emptive therapy. It identifies HSCT recipients at higher risk of reactivation based on the strength and timing of these responses, allowing for targeted monitoring and intervention. This assessment also evaluates the effectiveness of antiviral or adoptive T cell therapies in suppressing reactivation and helps identify recipients at risk for late onset of CMV disease, guiding ongoing management [48,49].

Focusing on T cell epitope composition in immune monitoring can benefit HSCT recipients. Comparing T cell epitope content measures the breadth and specificity of responses to CMV. These findings help evaluate the effectiveness of methods for detecting CMV-specific T cell immunity in transplant recipients, particularly HSCT patients [50,51]. This information can guide HSCT recipients in selecting the best immune-monitoring strategies and inform clinical decisions regarding prophylactic or pre-emptive therapy.

Research shows a strong link between empiric anti-CMV therapy and CMV reactivation in CMV-positive allogeneic HSCT recipients. Clinical symptoms often drive the start of anti-CMV therapy, rather than immunological monitoring results. However, immunological surveillance is still important. It helps assess CMV-specific T cell immunity and predict reactivation [48,52]. This monitoring can identify high-risk patients and suggest pre-emptive therapy before symptoms appear. Optimizing the timing and duration of anti-CMV therapy based on immune data and clinical factors is recommended. This approach may reduce reactivation risk while minimizing unnecessary treatment and side effects.

These studies collectively emphasize the importance of immune monitoring as a central component of CMV management in HSCT recipients, aiding in early detection, risk stratification, and personalized therapeutic interventions.

## 6. Immune Reconstitution in Haploidentical HSCT

HLA-haploidentical donors share a single HLA haplotype with transplant recipients, usually receive aggressive immunosuppression to prevent GVHD, and, in turn, are always at high risk of CMV infection [53,54]. Though there may be higher rates of CMV reactivation in haplo-HSCTs compared to HLA-matched transplants, the incidence of CS-CMVi is lower as evidenced by studies due to memory inflation in the presence of CMV reactivation [3,22,53]. It has been observed that antiviral prophylaxis delays CMV reactivation and in turn delays immune reconstitution leading to CS-CMVi post-cessation of antiviral prophylaxis [54,55,56]. Even with the recently introduced antiviral prophylaxis agent letermovir, delayed immune reconstitution is evident [56,57,58]. Hence, immune monitoring along with viral surveillance outweighs antiviral prophylaxis in high-risk transplant recipients. Sometimes, intensified immunosuppression in T-cell-depleted HSCT may delay CMV immune reconstitution in the early post-transplant period, and in this scenario immune recovery may be boosted by adoptive T cellular therapy in the event of CS-CMVi [22,45,53]. Henceforth, immune monitoring is to be considered essential in transplant protocols, as it not only helps in understanding the dynamics of immune reconstitution but also helps in identifying the need for adoptive T cell therapy [59].

## 7. Pre-Transplant Immune Monitoring

**Figure 3:**
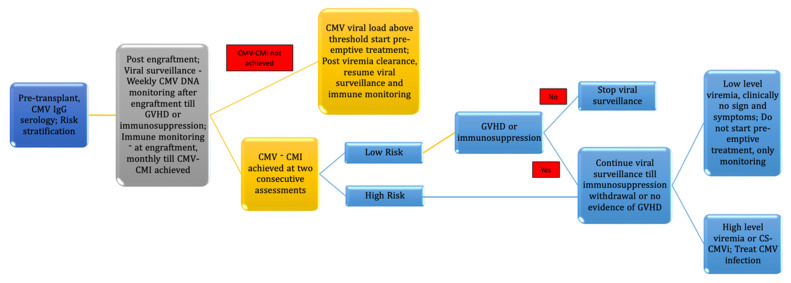
Proposed Algorithm for CMV surveillance and immune monitoring in HSCT.

As per conventional criteria and recommended guidelines, transplant centers follow the protocol for viral surveillance only after engraftment, as recipients are lymphopenic before engraftment. In parallel with this protocol, a few centers have looked into CMI only in the post-transplant period. It has been hypothesized that pre-existing CMV-specific CMI hastens the immune recovery in the post-transplant period by acting as a booster for donor-derived antigen-experienced T cells. However, studies by Seo et al. and Bae et al. have failed to prove the hypothesis [37,60]. Hence, monitoring CMV-specific pre-transplant immune monitoring may not be required. Finally, the advantages of immune monitoring for CMV in HSCT have been summarized in Table 3 and the proposed algorithm for immune monitoring is depicted in Figure 3.

## 8. Conclusions

Immune monitoring may help in risk stratification of transplant recipients who may progress from CMV reactivation to CS-CMVi. The implementation of this approach in clinical practice may help in reducing the need for periodic CMV DNAemia monitoring and unnecessary or prolonged antiviral therapy in recipients who mount high CMV-CMI and thus may have a self-limited reactivation. Thus, in the era of precision medicine, it is imperative to integrate CMV-specific cellular immune monitoring in the standard protocols and algorithms for the care of transplant recipients.

## Figures and Tables

**Table 1:** Various tests used for diagnosis of HCMV infection.

S. No.	Diagnostic Test
1.	**Serology:** Antibody detection of anti-CMV immunoglobulin (IgM and IgG), does not indicate active viral replication.
2.	**Viral culture:** Isolation of virus from multiple samples, including whole blood, plasma, sterile body fluids, urine, and biopsy using tissue cultures that use human embryonic fibroblast cells. However, detection of CMV in culture only indicates the presence of the virus or shedding of the virus and does not confirm active CMV disease. Moreover, these methods are time-consuming, lack sensitivity, and are rarely used nowadays.
3.	**Histopathology:** Histopathology is the gold standard for the diagnosis of tissue-invasive CMV disease by detecting cytomegalic cells and viral-specific inclusion bodies in a tissue biopsy specimen but requires invasive procedures like bronchoscopy or endoscopy to obtain a tissue sample.
4.	**Antigen detection:** This involves the detection of pp65 in peripheral blood leucocytes by using specific fluorescently labelled monoclonal antibodies in peripheral blood polymorph nuclear leukocytes. This technique is sensitive, specific, and quantitative. The antigen detection can also be used for monitoring therapeutic response. However, being labour-intensive is not used widely.
5.	**Molecular assay:** PCR is highly sensitive as it can detect minute amounts of nucleic acid in various clinical samples, determine viral load, and can be used for therapeutic monitoring.

**Table 2:** Studies highlighting the role of CMV cell-mediated immune reconstitution on CMV reactivation in HSCT.

Studies	Study Design	Study Population	CMV Cell-Mediated Immune Assessment	Method for CMV Assessment	Results
Chemaly et al., React Study, 2020 [36]	Multicentric 13 centers USA, Canada, UK, Sweden	241 adult patients (>18 years) Allogenic HSCT R^+^	Pre-transplant: d = 14, Post-transplant: d +14, +28, every 15 d up to 6 months	CMV pp65 and IE1-specific ELISPOT assay	CMV-CMI, independent predictor of CS-CMVi (*p* = 0.04) CMV-CMI low in patients who experienced CS-CMVi (94%), and high CMV-CMI has less CS-CMVi (*p* < 0.0001)
Seo et al., 2021 [37]	Australia	52 pediatric patients Allogenic HSCT D^+^/R^−^ status: 45 D^−^/R^−^ status: 4 D^−^/R^−^ status: 3	Pre-transplant: once Post-transplant: 1, 2, 3, 4, 5, 6, 9, 12 months Monitoring terminated when CMV-CMI recovered at two consecutive evaluations	CMV pp65 and IE1-specific ELISpot assay	Pre-HSCT CMV-specific CMI > 5 SFC/2 × 10^5^ cells significant predictive factor CMV-CMI recovery post-HSCT, but not CS-CMVi; Recovery of CMV-CMI > 50 SFC/2 × 10^5^ cells Post-HSCT protective factor for CS-CMVi (aOR = 0.13; 95% CI = 0.22–0.71)
Lilleri et al., 2012 [24]	Italy	131 adult and pediatric patients Allogenic HSCT D^+^/R^+^ status: 51 D^−^/R^+^ status: 38 D^+^/R^−^ status: 42	Post-transplant— monthly until day 180, then every 3 months until the detection of CMV-specific CD4^+^ and CD8^+^ T cells	Flow cytometric analysis for CMV-specific CD4^+^ and CD8^+^ T cells producing IFN-**γ** and IL-2	In the absence of GvHD, virus-specific T cell immune response remained stable after recovery, and the patients did not require treatment for CMV reactivation after achieving protective immunity
Nesher et al. [38]	USA	63 adult patients Allogeneic HSCT D^+^/R^+^ status: 41 D^−^/R^+^ status: 22	Pre-transplant Post-transplant d 30 (±7 d), d 60 (±7 d), and d 100 (±14 d)	CMV-specific IE-1 and pp65 antigens ELISpot assay	Thresholds (50 spots/250,000 cells for IF-1; 100 spots /250,000 cells for pp65) identified patients who were protected against CMV infection CMV-specific ELISpot response above the determined thresholds only significant factor for preventing CMV reactivation (aHR—0.21; 95% confidence interval, *p* = 0.046)
Tey et al., 2013 [34]	Australia	41 adult patients (>18 years) Allogenic HSCT D^+^/R^−^ status: 14 D^−^/R^−^ status: 24 D^−^/R^−^ status: 3	Post-transplant weekly between 3 and 14 weeks, 6 and 12 months	CMV-specific peptides pp65, IE1, epitopes from pp50, IE2 and gB; QuantiFERON CMV assay	The median time to stable CMV-specific immune reconstitution was 59 days, incidence of CMV reactivation lower in patients who developed this than those who did not (27% versus 65%; *p* = 0.031) Failure to reconstitute CMV-specific immunity soon after the onset of CMV viremia associated with higher peak viral loads (5685 copies/mL versus 875 copies/mL; *p* = 0.002)
Naik et al. [39]	USA	23 pediatric patients >2 years Allogenic HSCT	Pre-transplant: once Post-transplant: 1, 2, 3, 4, 5, 6, 9, 12 months	CMV pp65, IE1-specific IFNg ELISpot Longitudinal assessment of CD3^+^ T cells by FACS	Recipients with CD3^+^ counts >300 cells/microL exhibited potent functionally protective levels of CMV-directed T cell activity (defined as >30 antigen-specific SFCs/5 × 10^5^), rapidly cleared viral reactivation
Lee et al. [35]	Republic of Korea	33 pediatric patients Allo-HSCT D^+^/R^+^ status: 16 D^+^/R^−^ status: 7 D^−^/R^−^ status: 2	Baseline at 4 weeks post-HSCT Every time with CMV antigenemia (pp65) till the end of CMV treatment, 7 d after CMV antigenemia becomes negative	QuantiFERON CMV assay	Patients who had positive QF-CMV results after CMV reactivation had no recurrent infections thereafter, patients with indeterminate or negative results had recurrent CMV infections. Established CMV-specific T cell immunity following initial CMV infection to prevent recurrent CMV infection episodes

Abbreviations: CMV—Cytomegalovirus; HSCT—Hematopoietic stem cell transplant; CMI—cell-mediated immunity; D—Donor; R—Recipient; d—day; CS-CMVi—clinically significant CMV infection; FACS—Fluorescence-activated cell sorting; QF—QuantiFERON; SFC—Spot-forming cell; IFN-γ—Interferon-gamma; IL—Interleukin; GvHD—Graft versus host disease; CD—Cluster differentiation; ELISpot—Enzyme-linked immunosorbent spot assay.

**Table 3:** Utility of CMV immune monitoring in HSCT recipients.

1	Predict CMV reactivation
2	Shorten the duration of pre-emptive therapy
3	Predict those who could clear the virus spontaneously
4	Predict those who could contract an end-organ disease
5	Predict recurrent CMV reactivation following the first reactivation episode
6	To determine the need for adoptive T cell therapy

## Data Availability

The data will be made available by the corresponding author upon reasonable request.
